# A Novel mRNA Level Subtraction Method for Quick Identification of Target-Orientated Uniquely Expressed Genes Between Peanut Immature Pod and Leaf

**DOI:** 10.1007/s12575-009-9022-z

**Published:** 2009-12-24

**Authors:** Ning Wu, Kanyand Matand, Sonya Williams

**Affiliations:** 1 Center for Biotechnology Research and Education, Langston University, P.O. Box 1730, Langston, OK 73050, USA

**Keywords:** Peanut, subtraction, gene discovery, cDNA library, hybridization, mRNA

## Abstract

Subtraction technique has been broadly applied for target gene discovery. However, most current protocols apply relative differential subtraction and result in great amount clone mixtures of unique and differentially expressed genes. This makes it more difficult to identify unique or target-orientated expressed genes. In this study, we developed a novel method for subtraction at mRNA level by integrating magnetic particle technology into driver preparation and tester–driver hybridization to facilitate uniquely expressed gene discovery between peanut immature pod and leaf through a single round subtraction. The resulting target clones were further validated through polymerase chain reaction screening using peanut immature pod and leaf cDNA libraries as templates. This study has resulted in identifying several genes expressed uniquely in immature peanut pod. These target genes can be used for future peanut functional genome and genetic engineering research.

## 1. Background

Subtraction technology has sparked great interests in genomic field research because of its potentially broad applications in global gene discovery and efficiency in specific differentially expressed gene identification [1]. It is potentially a useful tool for gene expression profiling in new species [2], enhancing our understanding of many organismal molecular and physiological mechanisms [3-5], and facilitating the development of diagnostic markers and therapeutic drugs [6,7]. Most current protocols or commercially available kits apply relative subtraction, which is primarily based on the transcript variance of differentially expressed genes among investigated materials [7-9]. However, limited protocols that have highlighted absolute subtraction strategies were all coupled with polymerase chain reaction (PCR) for subsequent subtractive library constructions [10,11]. Therefore, resulting final subtracted libraries were generally of larger clone capacity mixtures of primarily differentially expressed genes. This limits rapid and more efficient detections of uniquely expressed target gene(s) within a defined biological material or process [5,7]. Such challenge coupled generally with the larger clone capacity of the first subtraction library had inspired current subtraction protocol modifications [4,12], among which some have involved a few to extended serial subtractions [5,8,13], whereas the others had combined both subtraction and microarray hybridization [4,7,9] for determining target specific genes. However, this usually is tedious and entails technical complexity and increased reagent and labor costs while also extending the experimental period.

The present study describes a novel subtraction approach based on mRNA level hybridization, which consists of detracting all across-tissue expressed genes in a single round subtraction, leaving only target-specific uniquely expressed genes. This method takes into account the challenges described earlier while simplifying the whole subtraction process. It was inspired from our previously reported paired library absolute subtraction technology [6] and is currently applied on our ongoing peanut (*Arachis hypogaea* L.) genomics research. In this investigation, we had hypothesized identifying target genes expressed *only* in peanut immature pod by subtracting, in a single round, all expressed genes common to both tissues.

## 2. Materials and Methods

### 2.1. Tissue Sample Collection

Immature peanut plants (Spanish breeding line, Tamspan 90) from seeds, kindly provided by Texas A&M Experimental Station and cultured in Langston University's greenhouse, were employed in this study. Two types of peanut plant organs, immature pod and leaf, were collected by immediate immersing in liquid nitrogen after removing it from the whole plant and stored at -80°C until the next procedure of total RNA isolation.

### 2.2. Total RNA Extraction and mRNA Purification

Total RNAs of above tissues were isolated by using Plant RNA Reagent (Invitrogen, Carlsbad, CA, USA) according to the manufacturer's instructions. The total RNA quality and quantity were determined by using SmartSpec™ 3000 spectrophotometer (Bio-Rad Laboratories, Inc., Hercules, CA, USA) at the ultraviolet absorbance of 260 and 280 nm, respectively. In addition, 1% agarose gel electrophoresis at 60 V with 50% of formamide in loaded samples was applied for RNA quality examination. Further, messenger RNAs of both tissues were purified using in-house-developed biotinylated oligo-dT magnetic particle technology. Purified mRNAs were subjected to quality and quantity determination by spectrophotometer.

### 2.3. Common Genes Subtraction

**Figure 1:**
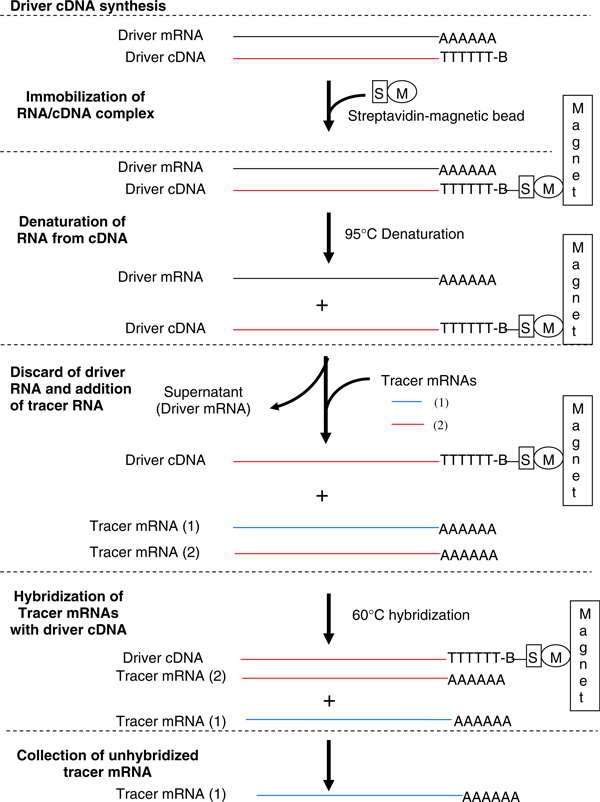
**General scheme applied for identifying peanut immature pod-specific genes (tracer mRNA (1)) after a single round subtraction**. *B* biotin, *S* streptavidin, *M* magnetic bead.

The subtraction procedures are illustrated in Figure 1. The expressed genes in immature pod and leaf tissues are defined, in these procedures, as *tracers* and *drivers*, respectively.

#### 2.3.1. Synthesis of Single-Stranded cDNA Drivers

Totally, 1.2 μg of peanut leaf mRNA was mixed with 200 ng of biotinylated oligo-dT primer ([B-]GACT[-B]AGT[-B]T[-B]CTAGATCGCGAGCGGCCGCCTGA(T)_15_) ([B-]: biotinylated nucleotide) at the volume of 10 μl. After 10 min incubation at 70°C, the mRNA/primer mixture was transferred to 45°C and mixed with 5× first-strand buffer, 25 mM MgCl_2_, 10 mM deoxyribonucleotide triphosphate (dNTP) mix, and ImProm-II™ Reverse Transcriptase (Promega, Madison, WI, USA) at the total volume of 40 μl. The reaction mixture was then incubated at 45°C for 65 min. The reaction volume was increased to 150 μl by adding Tris–EDTA (TE) buffer before mixing it with 160 μl of TE-washed streptavidin-coated magnetic beads (1.1 mg/ml; New England BioLabs, Ipswich, MA, USA). The mixed suspension was then incubated at room temperature for 60 min with constant rotating and then inserted into a magnet for 2 min before removing the supernatant. Isolated beads were washed three times by adding 100 μl of wash buffer (10 mM Tris-HCl (pH 7.5), 1 mM EDTA, 500 mM NaCl) and re-inserted into the magnet for 2 min each time. Cleaned beads were resuspended in 100 μl of wash buffer and incubated at 95°C for 5 min followed by inserting in the magnet for 2 min. The supernatant was discarded, and the same step was repeated once to thoroughly remove leaf mRNAs. The synthesized single-stranded cDNA drivers were resuspended in 30 μl of freshly prepared 5× first-strand buffer, 25 mM MgCl_2_, and diethylpyrocarbonate-treated water and ready for subtraction hybridization.

#### 2.3.2. Subtraction Hybridization of mRNA Tracers and cDNA Drivers

About 600 ng of immature pod mRNAs (tracer) in 10 μl volume was prepared for subtraction hybridization with premade single-stranded cDNA drivers at an original tracer/driver mRNA weight ratio of 1:2. The tracer mRNAs were mixed with magnetic beads attached with first-strand cDNA drivers and then incubated at 60°C for 65 min for tracer/driver hybridization followed by transferring reaction mixture to magnet for 5 min. The supernatant was carefully collected into a fresh tube and reinserted into magnet for another 5 min. The supernatant that contained unhybridized tracer mRNAs was collected carefully for the following subtraction library construction process.

### 2.4. Subtracted Library Construction

Unhybridized tracer mRNAs from the previous step were primed by adding 200 ng of biotinylated oligo-dT primer with an integrated Not I restriction site, 10 mM dNTP mix, and ImProm-II™ Reverse Transcriptase at 45°C for 65 min to synthesize the first-strand cDNAs in the total volume about 40 μl. The second-strand cDNAs were synthesized by adding 5× second-strand buffer, 10 mM dNTP, 42 U of *Escherichia coli* DNA polymerase, 12 U of *E. coli* DNA ligase, and 2 U of RNase H to the first-strand reaction mixture to bring to the final volume of 150 μl. The mixture was incubated immediately at 16°C for 2 h before adding 10 U of T4 DNA polymerase for 5 min additional incubation. Streptavidin-coated magnetic beads (1.1 mg/ml) were applied again to purify synthesized double-stranded cDNAs with three times washes. The double-stranded cDNAs were released from the magnetic beads by Not I (75 U) restriction digestion at 37°C for 1 h followed by the same volume phenol/chloroform/isoamyl alcohol (25:24:1) extraction and precipitation using 7.5 M sodium acetate, glycogen (20 μg/μl), and ice-cold absolute ethanol at -20°C overnight. The precipitated cDNAs were ligated to pretreated pCMVSPORT6 (with Not I and blunt end cloning sites) by adding 5 U of T4 DNA ligase and incubating at room temperature for 3 h. The cloned cDNAs were electroporated to ElectroMax DH10B competent cells (Invitrogen, Carlsbad, CA, USA). Electrotransformed cells were recovered in 2 ml of Luria–Bertani (LB) medium by incubating at 37°C with gently shaking for 1 h. Three hundred microliters of cells from subtraction library was removed for plating assays (100 μl for each assay) to determine subtraction library capacity before 1 ml of transformed cells was inoculated in 10 ml of LB/Amp medium for overnight amplification. Glycerol was added to both unamplified and amplified subtraction libraries to the final concentration of 20%. Both libraries were stored at -80°C for future applications.

### 2.5. Target Clone Sequencing and Primary Bioinformatics Analysis

Three hundred microliters of unamplified subtracted cDNA library was plated on LB/Amp plates with 100 μl on each and incubated at 37°C overnight. The number of total colony-forming units (cfu) was calculated to determine the subtracted library capacity. The colonies were randomly picked for individual clone amplification. Plasmid DNAs were purified for DNA sequencing. The sequencing operation was down by using PT-100 Thermocycler (Bio-Rad Laboratories, Inc., Hercules, CA, USA), GenomeLab™ Dye Terminator Cycle Sequencing Quick Start Kit, and CEQ8800 Genetic Analysis System (BECKMAN COULTER, Fullerton, CA, USA) following the manufacturer's instructions. All resulted sequences were analyzed by BLASTN (http://www.ncbi.nlm.gov/blast ) against GenBank "Nucleotide collection" (nr) and "Expressed sequence tags" (est) databases and clustered into individual gene groups based on the sequences matched percentage and the similarities (both must be 90% or above).

### 2.6. Target Gene Primer Design and PCR Screening Experiments

According to the sequence analysis results, the potential highly differential or specific expressed target genes were determined and the related PCR primers were designed by using Primer3 (http://frodo.wi.mit.edu/cgi-bin/primer3/primer3_www.cgi) software and synthesized for each candidate gene. A prevalent peanut allergen gene, Ara h 1 (GenBank accession number AF432231), was selected as the control for PCR experiments. The templates for PCR experiments were the in-house-synthesized immature peanut pod and leaf cDNA libraries. The PCR reactions were performed using GoTaq Green Master Mix (Promega, Madison, WI, USA) following the manufacturer's instructions. The reactions results were analyzed by 2% agarose gel electrophoresis.

## 3. Results and Discussion

### 3.1. Peanut Immature Pod Subtracted Library Capacity and Clone Sequencing

The unamplified subtracted library resulting from immature pod processing contained 320 cfu that were determined by calculation of colonies from plating assays. This library clone capacity was much lesser than those of serial subtraction [5,13] or standard PCR-based cDNA subtraction protocols, which often generate libraries of capacities ranging from a few thousands to more than 10,000 clones [4,8,11,12]. Totally, 46 clones from plating assays were randomly selected for 5'-end single-pass DNA sequencing.

Sequencing data analysis showed that the redundancy of the subtracted library was about 20%. Of the 46 sequenced clones, 12 (26%) were distributed into three gene clusters. The remaining clones (74%) were all singletons and represented 34 individual gene clusters. The clone redundancy occurrence in subtracted library was greatly minimized due to the significant reduction of common clone population during the subtraction process. The clustered gene groups and related BLASTN analysis results are presented in Table 1. It showed that 23 clones represented by 14 gene clusters matched homologs of peanut nucleotide sequences of six known functional genes and eight expressed sequence tag (EST) sequences. Based upon GenBank records, all matched homolog sequences originated from peanut immature pod and/or seed cDNA libraries. None of them originated from peanut leaf tissue. The additional 23 gene clusters showed no significant matches with records in both "*nr*" and "*est*" databases and were therefore defined as novel genes. Subtraction is a powerful technology, especially in studies aimed at large-scale gene discoveries by removing unwanted genes thoroughly from the targeted libraries and by progressively enriching the libraries with the novel and rarer genes [2,8].

**Table 1:** BLASTN search result for 37 individual gene clusters from peanut immature pod subtraction cDNA library

Cluster ID	Clone number	Homolog description	Sequence matched %	Sequence identity %
1	1	nr: no match		
		EST: no match		
2	1	nr: no match		
		EST: Z6 Peanut (Luhua14) seeds full length cDNA Library *Arachis hypogaea* cDNA 5' similar to unknown protein, mRNA sequence	99	100
3	1	nr: no match		
		EST: no match		
4	1	nr: no match		
		EST: no match		
5	1	nr: no match		
		EST: no match		
6	1	nr: no match		
		EST: no match		
7	1	nr: no match		
		EST: NN34 Peanut (Luhua14) seeds full length cDNA Library *Arachis hypogaea* cDNA 5' similar to desiccation-related protein, mRNA sequence	99	94
8	1	nr: no match		
		EST: UTPPI008_C01 UTPP *Arachis hypogaea* cDNA clone UTPPI008_C01 5', mRNA sequence	100	98
9	1	nr: no match		
		EST: no match		
10	1	nr: *Arachis hypogaea* (clone P17) Ara h I mRNA, complete cds	100	100
		EST: 16 Peanut (Luhua14) seeds full length cDNA Library *Arachis hypogaea* cDNA 5' similar to conarachin, mRNA sequence	100	100
11	1	nr: no match		
		EST: no match		
12	1	nr: no match		
		EST: no match		
13	1	nr: no match		
		EST: no match		
14	1	nr: no match		
		EST: no match		
15	1	nr: no match		
		EST: no match		
16	3	nr: *Arachis hypogaea* allergen Arah3/Arah4 gene, complete cds	100	100
		EST: 1X56 Peanut (Luhua14) seeds full length cDNA Library *Arachis hypogaea* cDNA 5' similar to storage protein, mRNA sequence	100	100
17	1	nr: no match		
		EST: no match		
18	1	nr: *Arachis hypogaea* oleosin 1 mRNA, complete cds	100	100
		EST: UTPPI011_G11 UTPP *Arachis hypogaea* cDNA clone UTPPI011_G11 5', mRNA sequence	100	100
19	1	nr: no match		
		EST: no match		
20	1	nr: no match		
		EST: no match		
21	1	nr: no match		
		EST: no match		
22	3	nr: *Arachis hypogaea* seed storage protein SSP1 mRNA, partial cds	94	100
		EST: 1T61 Peanut (Luhua14) seeds full length cDNA Library *Arachis hypogaea* cDNA 5' similar to seed storage protein SSP2, mRNA sequence	94	100
23	6	nr: *Arachis hypogaea* conglutin mRNA, complete cds	95	98
		EST: 1E12 Peanut (Luhua14) seeds full length cDNA Library *Arachis hypogaea* cDNA 5' similar to conglutin protein, mRNA sequence	97	98
24	1	nr: no match		
		EST: no match		
25	1	nr: no match		
		EST: no match		
26	1	nr: no match		
		EST: no match		
27	1	nr: no match		
		EST: no match		
28	1	nr: *Arachis hypogaea* allergen II gene, partial cds	100	100
		EST: NN91 Peanut (Luhua14) seeds full length cDNA Library *Arachis hypogaea* cDNA 5' similar to 2S protein 1, mRNA sequence	100	100
29	1	nr: no match		
		EST: no match		
30	1	nr: no match		
		EST: no match		
31	1	nr: no match		
		EST: 1S91 Peanut (Luhua14) seeds full length cDNA Library *Arachis hypogaea* cDNA 5' similar to histone H2B, mRNA sequence	90	99
32	1	nr: no match		
		EST: no match		
33	1	nr: no match		
		EST: U16 Peanut (Luhua14) seeds full length cDNA Library *Arachis hypogaea* cDNA 5' similar to 2S protein 2, mRNA sequence	99	98
34	1	nr: no match		
		EST: UTPPI012_E03 UTPP *Arachis hypogaea* cDNA clone UTPPI012_E03 5', mRNA sequence	91	98
35	1	nr: no match		
		EST: UTPPI002_H03 UTPP *Arachis hypogaea* cDNA clone UTPPI002_H03 5', mRNA sequence	100	99
36	1	nr: no match		
		EST: no match		
37	1	nr: no match		
		EST: UTPPI002_F08 UTPP *Arachis hypogaea* cDNA clone UTPPI002_F08 5', mRNA sequence	95	100

*UTPP* USDA-Tifton Peanut Immature Pod cDNA Library

**Figure 2:**
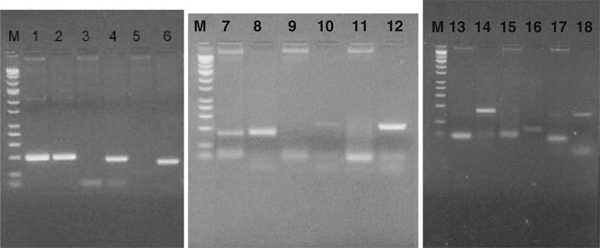
**PCR screening test for peanut immature pod-specific genes**. PCR templates: peanut leaf cDNA library (*lanes 1*, *3*, *5*, *7*, *9*, *11*, *13*, *15*, *17*) and peanut immature pod cDNA library (*lanes 2*, *4*, *6*, *8*, *10*, *12*, *14*, *16*, *18*). PCR products: control gene (~194 bp; *lanes 1* and *2*); candidate genes that are only expressed in peanut pod: cluster 7 (~173 bp; *lanes 3* and *4*), cluster 31 (~152 bp; *lanes 5* and *6*), cluster 33 (~233 bp; *lanes 9* and *10*), cluster 15 (~218 bp; *lanes 11* and *12*), cluster 11 (~170 bp; *lanes 13* and *14*), cluster 8 (~80 bp; *lanes 15* and *16*); candidate genes that are more highly expressed in peanut immature pod than leaf: cluster 23 (~152 bp; *lanes 7* and *8*), cluster 24 (~160 bp; *lanes 17* and *18*). *M* DNA marker.

It is noteworthy that the content of our subtracted library highlighted two types of genes: (a) genes that were expressed exclusively in peanut pod and (b) those that expressed in both pod and leaf tissues but with a distinctly *higher expression level* in immature pod than leaf (Figure 2). This enabled us to identify the genes that were either unique to immature pod or functionally more expressed in this tissue.

### 3.2. Identification of Unique Expressed Genes in Peanut Immature Pod

All resulting individual gene clusters were subjected to further PCR experimental validation by using in-house-made peanut immature pod and leaf cDNA libraries as templates to identify the unique expressed genes in immature pod. Totally, 25 pairs of primers, each representing a single gene cluster, were designed with the PCR product ranging from 80 to 237 bp long. The rest of gene clusters failed the primer designing program due to the lack of preferable primer sequences within the target clones. A prevalent peanut allergen gene, Ara h 1, was selected as the control for PCR experiments. The PCR screening resulted in identifying six target genes, whose expressions were unique to immature pod. Those six clones represented six individual gene clusters (cluster Nos. 7, 8, 11, 15, 31, and 33) and showed significant PCR products only in the reactions using immature pod cDNAs as the templates. There was no evidence of PCR products in the reactions of all six clones where peanut leaf cDNAs were used as templates (Figure 2). Based upon BLASTN results, all six target genes either matched with peanut immature pod/seed ESTs of unknown functions or had no matches in both "*nr*" and "*est*" databases. The databases showed no evidence of expression of any of those six candidate genes in any peanut plant tissues other than immature pod or seed. The PCR results provided solid evidence of the effectiveness of our approach in identifying expressed genes unique to peanut immature pod. The six candidate genes are considered as possible valuable targets for further genome functional studies on peanut biology and developmental mechanisms. These results could be used for applications in both basic and applied research in peanut and/or other legumes. Identified genes could be studied further for their structure, function, transcription control, regulation, etc. It could also be cloned in full-length format for transformation aimed at the crop improvement.

## 4. Concluding Remarks

This report outlines a novel and universal subtraction method of higher efficiency and accuracy. Its application reduces the library clone redundancy and capacity, while enhancing chances of quick and accurate identification of target-orientated specific genes *through a single round subtraction.* Most importantly, when comparing it to the other current subtraction protocols, the present approach is simple and cost, product, time, and labor effective. It can be performed in most conventional laboratories without requiring the purchasing of expensive kits and/or pieces of equipment. The highlights of this protocol include (a) its great success for driver preparation and subtractive hybridization *using just tiny amounts of mRNAs*, when comparing it to most current protocols that require huge amount of mRNAs for the same purpose [14]. (b) As compared with previously reported protocols that apply paramagnetic oligo (dT) beads as solid base for subtractive hybridization [10,11], streptavidin-coated magnetic beads provide the strongest and irreversible binding capability to immobilize the drivers, which ensures the elimination of hybridized common mRNAs. (c) The protocol does not require PCR cloning for subtractive library construction. This is beneficial because it prevents developing subtractive libraries with larger capacities of clones as previously reported [4,5,8,11-13]. (d) There is no need for radioisotopes and/or some related specialized enzymes such as [α-^32^P]dCTP [11,14] and terminal transferase [11]. Further, the method ensures that subtraction experiments can be carried out *fully* in 1 day. Thus, its application could potentially be more appealing to most conventional research laboratories, especially those with radioactive safety concern and/or limited labor for conducting concomitantly several experiments including those requiring tedious subtractive investigations. (e) The protocol involves fewer steps and requires only general knowledge and skills in handling RNA materials. Finally, it is hoped that this report could simplify and lead to a widespread application of subtraction in genomic research, novel gene discovery, and target gene identification across species.
